# A data-driven high-accuracy modelling of acidity behavior in heavily contaminated mining environments

**DOI:** 10.1038/s41598-025-14273-9

**Published:** 2025-09-30

**Authors:** Ana Barroso, Teresa Maria Valente, Amélia Paula Marinho Reis, Isabel Margarida Horta Ribeiro Antunes

**Affiliations:** 1 ICT – Institute of Earth Sciences, Pole of University of Minho, University of Minho, Braga, Portugal; 2 GEOBIOTEC, Geosciences Department, University of Aveiro, Campus Universitário de Santiago, Aveiro, 3810-193 Portugal

**Keywords:** Hydrochemistry, PTE concentration, Statistic tools, Multiple linear regression, Iberian pyrite belt, Trimpancho, Environmental sciences, Hydrology

## Abstract

Accurate estimation of water acidity is essential for characterizing acid mine drainage (AMD) and designing effective remediation strategies. However, conventional approaches, including titration and empirical estimation methods based on iron speciation, often fail to account for site-specific geochemical complexity. This study introduces a high-accuracy, site-specific empirical model for predicting acidity in AMD-impacted waters, developed from field data collected at the Trimpancho mining complex in the Iberian Pyrite Belt (Spain). Using multiple linear regression (MLR), a robust predictive relationship was established based on Cu, Al, Mn, Zn, and pH, achieving a coefficient of determination (R²) of 99.2%. The model significantly outperforms the standard Hedin method, with a lower mean absolute percentage error (13% vs. 29%). Results also reveal strong spatial and seasonal hydrochemical variability, underscoring the limitations of generalized acidity models in such environments. This work demonstrates the applicability of site-calibrated multivariate models as practical tools for enhancing acidity prediction in complex AMD systems.

**Supplementary Information:**

The online version contains supplementary material available at 10.1038/s41598-025-14273-9.

## Introduction

Understanding and accurately predicting acidity in contaminated aquatic systems is essential for assessing environmental risks, guiding remediation strategies, and protecting both ecosystems and human health. Acid mine drainage (AMD)—a persistent phenomenon in post-mining landscapes—presents a unique challenge due to its complex geochemistry and long-lasting impacts on water quality and human health^1–8^. AMD results from the oxidative weathering of sulfide minerals, such as pyrite (FeS₂), producing sulfuric acid and mobilizing PTEs into aqueous systems^1,9^.

Abandoned or poorly regulated mining sites, such as those found in the Iberian Pyrite Belt (IPB), provide striking examples of this environmental issue. The IPB is a globally recognized mining province where legacy contamination remains largely unmanaged^10,11^. The Trimpancho Mining Complex, located in the Spanish sector of the IPB, exemplifies the long-term environmental consequences of mine abandonment, including uncontrolled AMD generation and downstream pollutant transfer. Seasonal variations in hydrology further complicate the behavior of AMD-affected waters, influencing acidity levels and the transport of dissolved metals^12–14^.

Key indicators for evaluating AMD effects include pH, electrical conductivity, sulfate, and dissolved metals^15^. Among these, acidity plays a central role in driving metal mobility and ecosystem toxicity. Accurate acidity prediction is critical for designing site-specific interventions such as passive or active treatment systems, selecting neutralizing agents, and anticipating long-term environmental risks^16–18^. Traditional titration-based acidity measurements, particularly the hot acidity method^19^, provide robust quantification but are labor-intensive and impractical for broad-scale monitoring. Calculated acidity methods offer a more accessible alternative, relying on field-based measurements of pH and metal concentrations^20–23^. However, these models often overlook the complex geochemical interactions characteristic of AMD systems.

Recent advances emphasize the importance of integrating hydrochemical field data with multivariate statistical tools to improve process-based understanding and model performance^24,25^. Acidity generation involves the interplay of amphoteric metal hydrolysis, redox reactions, and mineral dissolution, all modulated by site-specific conditions. Therefore, generalized models calibrated under controlled laboratory settings often fail to capture the spatial and temporal variability inherent to real-world AMD environments. This highlights the need for tailored, data-driven approaches that reflect the unique hydroclimatic and geochemical context of each site.

This study addresses this critical gap by developing a site-specific, statistically grounded model to predict acidity in AMD-impacted waters from the Trimpancho mining complex. Field campaigns across multiple seasons were conducted to capture hydrochemical variability. By applying linear discriminant analysis and multiple linear regression, we identify the dominant geochemical drivers of acidity and assess their predictive power. The resulting framework enhances understanding of acidity generation under complex environmental conditions and supports more effective monitoring and remediation strategies. This research contributes to the broader discourse on environmental legacy pollution, demonstrating how field-based modeling can bridge geochemical complexity and management needs across interconnected environmental spheres.

## Materials and methods

### Study area

**Fig. 1:**
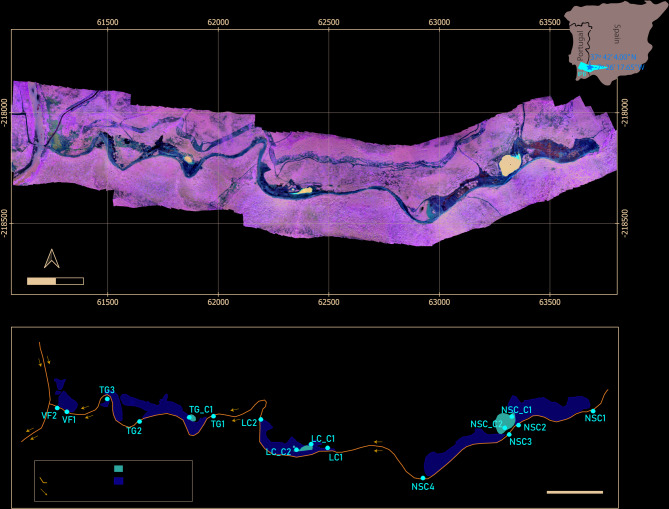
Overview of the Trimpancho mining complex. (**A**) Orthomosaic generated from a drone-based photogrammetric survey, showing the geographic location and extent of the study area. (**B**) Schematic representation of the mining areas and fixed water sampling points collected in the Trimpancho stream and pit lakes. *Seep* sampling points are not represented, as they are not fixed and could not be sampled consistently across all field campaigns.

The Trimpancho mining complex (Fig. 1A) is located in the Huelva Province, in the Western Region of the Spanish part of the IPB, one of the largest metallogenic provinces in the world^26–28^. This region is known for its volcanogenic massive sulfide (VMS) deposits, which contain a diverse range of minerals, including pyrite (FeS_2_), sphalerite (ZnS), chalcopyrite (CuFeS_2_), galena (PbS), arsenopyrite (FeAsS), and sulfosalts. The IPB, which spans the border between Portugal and Spain, faces ongoing challenges related to AMD, largely due to numerous abandoned mines characterized by waste materials exposed to weathering conditions [e.g ^14,29–31^].

The Trimpancho Complex is comprised of four abandoned mines: Volta Falsa (VF), Trimpancho Group (TG), La Condesa (LC), and Nuestra Señora del Carmen (NSC) (Fig. 1A and B)^32^. These mines ceased activity by the end of the 20th century without implementing remediation actions. The area includes three major acidic pit lakes and multiple waste deposits, collectively covering approximately 8 hectares^33^. The waste deposits, which are rich in sulfide materials, are located along the Trimpancho River, a tributary of the Chança River, which ultimately feeds into the Chança Dam—an important reservoir of freshwater for human consumption. The leachates discharged into the river drainage network can lead to the contamination of downstream watercourses and the degradation of water quality^34,35^.

The climate of the study area is classified as Csa according to the Köppen-Geiger climate classification, a hot-summer Mediterranean climate, characterized as semi-arid due to low precipitation rates^36^. The region has an annual precipitation of approximately 630 mm and an average annual temperature of 17.5 °C^37^. These hydroclimatic conditions promote water scarcity, requiring urgent, more effective management strategies for maintaining the region’s water resources.

### Water sampling and analytical methods

Three sampling campaigns were conducted to align with varying meteorological conditions, in a total of 16 water sample points (Fig. 1B). The first campaign occurred during a dry period in February 2022, while the second and third campaigns took place during rainy periods in October 2023 and March 2024, respectively. The sampling network was designed to capture the diverse hydrochemical environments across the study area. Surface water samples were collected at multiple locations along the Trimpancho stream to evaluate the effect of individual mining waste dumps (NSC1-NSC4, LC1-LC2, TG1-TG3, and VF1-VF2). Additionally, samples were taken from the three pit lakes (NSC_C1, NSC_C2, LC_C1, LC_C2, and TG_C1) and several water seepages (NCS1_seep, TG_seep, and TG3_seep). These seeps are observed during both wet and dry periods and are classified as ephemeral, as their occurrence is strongly influenced by short-term hydrological dynamics. They originate from the infiltration and subsurface migration of drainage water from adjacent waste dumps, subsequently discharging at the surface under specific conditions. During periods of intense or initial rainfall, seeps may develop due to the rapid saturation of superficial layers, whereas in dry periods, low or absent surface runoff, combined with evaporation and infiltration, can also promote the formation of isolated accumulation zones along the riverbed. These seepage zones act as localized discharge points and often exhibit distinct geochemical signatures due to limited mixing and short residence times. In total, 50 samples were collected across the three campaigns, providing the dataset for representing hydrological and chemical dynamics.

At each sampling point, 500 mL of water was collected and stored in a sterilized polyethylene container. An aliquot of 50 mL of the sample was filtered through 0.45 μm membranes and acidified with nitric acid to a pH below 2, preventing metal precipitation and bacterial growth. All water samples were kept cool (at 4 °C) in dark conditions and transported to laboratory analysis.

In situ water parameters, including pH and EC, were obtained using a multiparameter instrument (Thermo Scientific Model Orion Star A Series) with a pH electrode triode (Orion 9107BNM) and a conductivity cell (Orion 01310MD). In the laboratory, acidity and sulfate concentrations were determined using volumetric titration and turbidimetry methods (Standard Methods 2310 B and 4500-SO4 -2 E)^19^. Concentrations of selected elements (Fe, Al, As, Cu, Zn, Mn, Cd, Pb, Co, Ca, Mg, K) were analyzed by inductively coupled plasma optical emission spectrometry (ICP-OES) in filtered and acidified aliquots. Iron speciation was determined using the Standard 3500 D-phenanthroline method^19^.

All reagents used were of analytical grade or Suprapur quality (Merck, Darmstadt, Germany). Metals and arsenic analyses were performed at Activation Laboratory, Lda (Actlabs, Ancaster, ON, Canada), with duplicate samples and blanks included to assess precision, and accuracy with certified standards. The standard solution Merck AA Certified and Milli-Q water were used in the experiments.

### Statistical analysis

All variables were examined for the potential influence of outliers. The data normality was assessed using histograms, box plots, quantile-quantile plots, and the Shapiro-Wilk test. The results showed that the variables exhibited a non-normal distribution, at the 95% significance level. Although variable transformations, such as square root and logarithmic transformations, are commonly used to achieve normality [e.g.^38^], these did not significantly improve the distribution of the Trimpancho data. Therefore, the analysis was conducted using the original data set.

Two categorical variables were created to better characterize seasonal influences (meteorological conditions) and different spatial hydrochemical environments. The first variable was the weather conditions, corresponding to distinct surveys, which were categorized into two groups (wet season and dry season), and the second was the type of water source, classified into three groups (stream water, pit lakes water, and seeps water). The Mann-Whitney U and Kruskal-Wallis H non-parametric tests were used to determine differences between groups, with results interpreted based on rank differences. A *p*-value of less than 0.05 was considered statistically significant when testing the null hypothesis of no differences across the considered categories. Post-hoc pairwise multiple comparisons were then performed using the Bonferroni correction to identify significant differences between the sampling survey and water types^38^. Spearman’s rank correlation coefficients (Spearman’s rho) were calculated to identify potential relationships between the variables. Since all the variables failed the normality test (as previously noted), a non-parametric correlation coefficient was selected as the most appropriate approach.

#### Model development

Linear Discriminant Analysis (LDA), a supervised multivariate classification technique, was employed to determine the linear combinations of variables (discriminant functions) that best separate predefined groups—in this case, the different water types. The number of discriminant functions generated is equal to one less than the number of groups being compared^39^. To evaluate the model’s discriminative power, Wilks’ lambda was calculated, with statistical significance set at *p* < 0.005. Additionally, leave-one-out cross-validation was performed to assess model robustness and to reduce the risk of overfitting, thus enhancing the generalizability of the classification results^40^.

Principal Component Analysis (PCA) was applied to explore patterns and associations among geochemical variables, particularly to investigate how acidity relates to the distribution of potentially toxic elements (PTEs). Prior to PCA, four samples were identified as outliers based on the results of the LDA and exploratory data analysis. These samples were excluded to avoid distortion of the multivariate patterns, resulting in a final dataset of 46 samples. The objective of the PCA analysis was to identify potential associations between acidity and the other parameters analyzed. Due to the considerable number of variables (m = 18) in comparison to the number of samples (*n* = 46), it was necessary to exclude some variables from the PCA. In alignment with the study’s objectives, the following variables were selected: acidity, Al, Cu, Fe, Pb, As, Zn, Cd, Co, Mn, and pH. PCA is a dimensionality reduction technique that transforms correlated variables into a new set of orthogonal components—principal components—while preserving the maximum variance in the data^41^. These components are expressed as linear combinations of the original variables, enabling a clearer interpretation of complex geochemical data^42,43^.

Following PCA, stepwise Multiple Linear Regression (MLR) analysis was conducted to identify the key hydrochemical parameters strongly associated with acidity. Variables were entered or removed from the model based on the probability of F to enter (< 0.05) and to remove (> 0.10). The Durbin–Watson statistic was used to test for the presence of first-order autocorrelation among the residuals, ensuring the independence of observations and model validity^44,45^.

All statistical analyses—including univariate and bivariate statistics, normality testing (Shapiro-Wilk), group comparisons (Mann-Whitney U and Kruskal-Wallis H tests), correlation analyses, LDA, and MLR—were conducted using IBM SPSS Statistics (v.28). PCA was also performed using the AnDad software package (v.7.12), a freely available tool designed for exploratory multivariate analysis.

## Results and discussion

The hydrochemical characteristics of the water samples collected during the three sampling periods are summarized in Table 1. pH values ranged from 1.67 to 3.06, indicating extremely acidic conditions consistent with those found in other AMD-affected regions of the IPB [e.g.^14,32,46,47^]. Sulfate and acidity concentrations were notably high, reaching 171,639 mg/L and 125,250 mg/L of CaCO3, respectively. The CV revealed substantial heterogeneity among the parameters, with particularly high values for trace metals such as Fe, Cu, and Pb, indicating significant spatial and temporal variability. In contrast, pH exhibited a low CV, reflecting stable acidic conditions across sampling sites.

**Table 1:** Summary statistics for the analyzed water parameters.

Parameters	Minimum	Maximum	Mean	Median	Standard-deviation	Coefficient of variation (CV)
pH	1.67	3.06	2.49	2.64	0.30	12
EC	1,129	51,260	7,137	4,462	8,956	125
Acidity	238	125,250	6,811	1,635	21,816	320
Sulfate	482	171,639	10,489	3,538	30,688	293
Fe_(total)_	0.06	32,400	1,327	56.1	5,248	395
Fe^2+^	---	16,818	393	0.58	2,402	611
Fe^3+^	0.06	16,147	934	55.4	3,163	339
Al	31.4	8,530	548	208	1,565	285
As	0.03	8.85	0.58	0.03	1.51	261
Cd	0.003	3.30	0.22	0.05	0.55	255
Ca	12.5	421	123	94.4	83.0	67
Cu	1.98	1,370	647	12.1	224	346
K	0.20	10.0	1.97	1.15	1.98	100
Mg	42.6	10,800	714	291	1,837	257
Mn	2.17	358	42.4	26.5	64.0	151
Co	0.06	18.3	1.10	0.46	2.89	264
Zn	0.51	520	60.7	14.3	123	203
Pb	0.01	7.28	0.38	0.06	1.20	312

Note: EC is expressed in µS/cm, acidity is expressed in mg/L of CaCO3, sulfate is expressed in mg/L, the concentration of all elements is expressed in mg/L, and the CV is expressed as a percentage (%).

Temporal variations in water quality between wet and dry seasons were significant (*p* < 0.05), with greater variability during wet periods. Stream water exhibited dynamic conditions influenced by hydrological changes, while pit lakes showed more stable geochemistry. Seeps, often ephemeral, had the highest PTE concentrations due to limited dilution and short residence time.

**Fig. 2:**
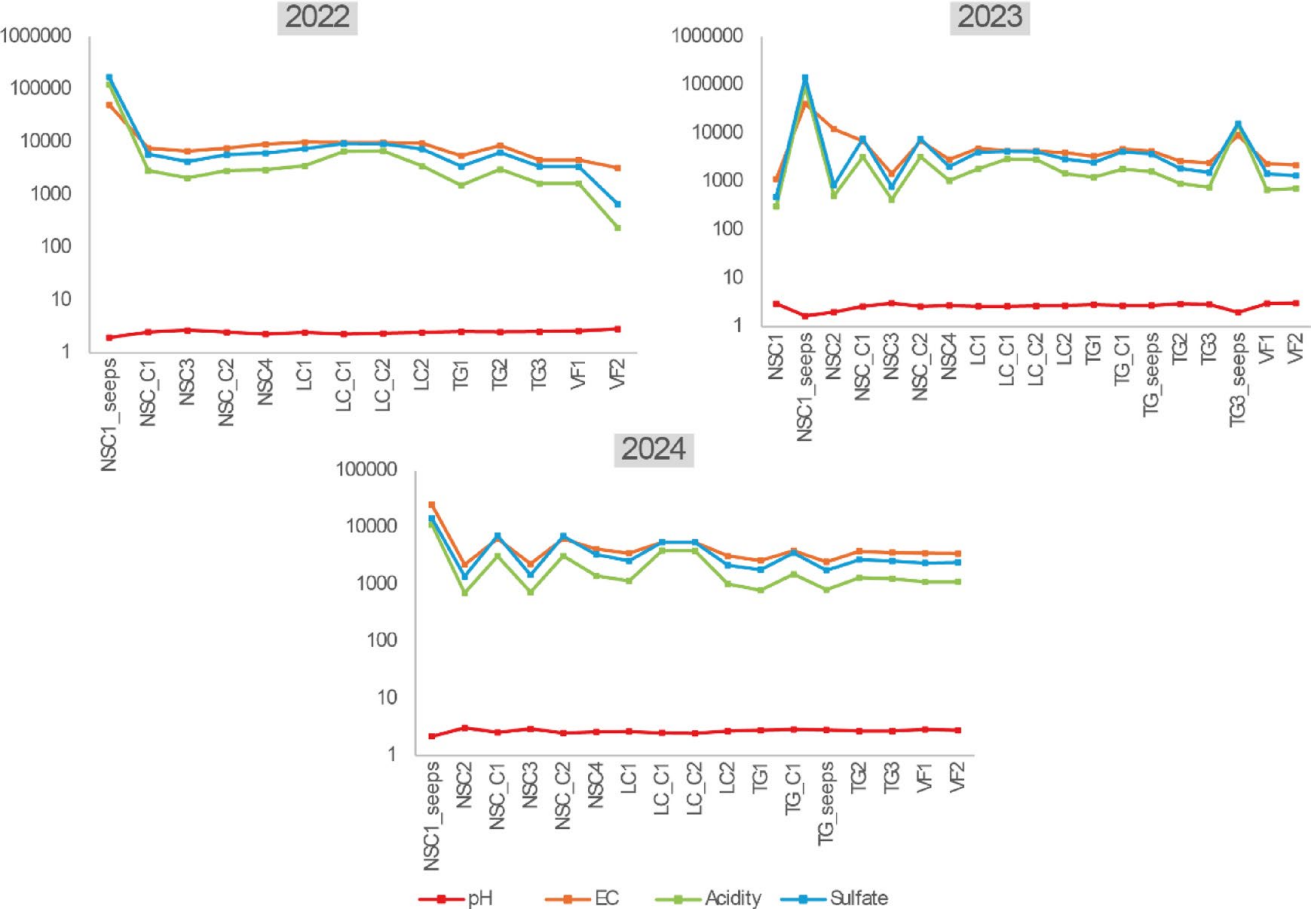
Spatial variation of water physicochemical properties (pH, EC, acidity, and sulfate) for the three sampling periods: February 2022, October 2023, and March 2024.

Figure 2 shows that stream water undergoes attenuation processes during dry periods, where evaporation promotes salt precipitation and hydrolysis reactions lead to the removal of metals via the formation of secondary minerals such as jarosite (KFe₃(SO₄)₂(OH)₆)^4,48,49^ For instance, the concentration of Fe exhibited a moderate decrease (495–1750 mg/L at the initial sampling point NSC and 13,6–28,5 mg/L at the final sampling point in the VF) along the stream, likely due to the natural attenuation processes associated with the precipitation of iron hydroxides^46,48,50^.

**Fig. 3:**
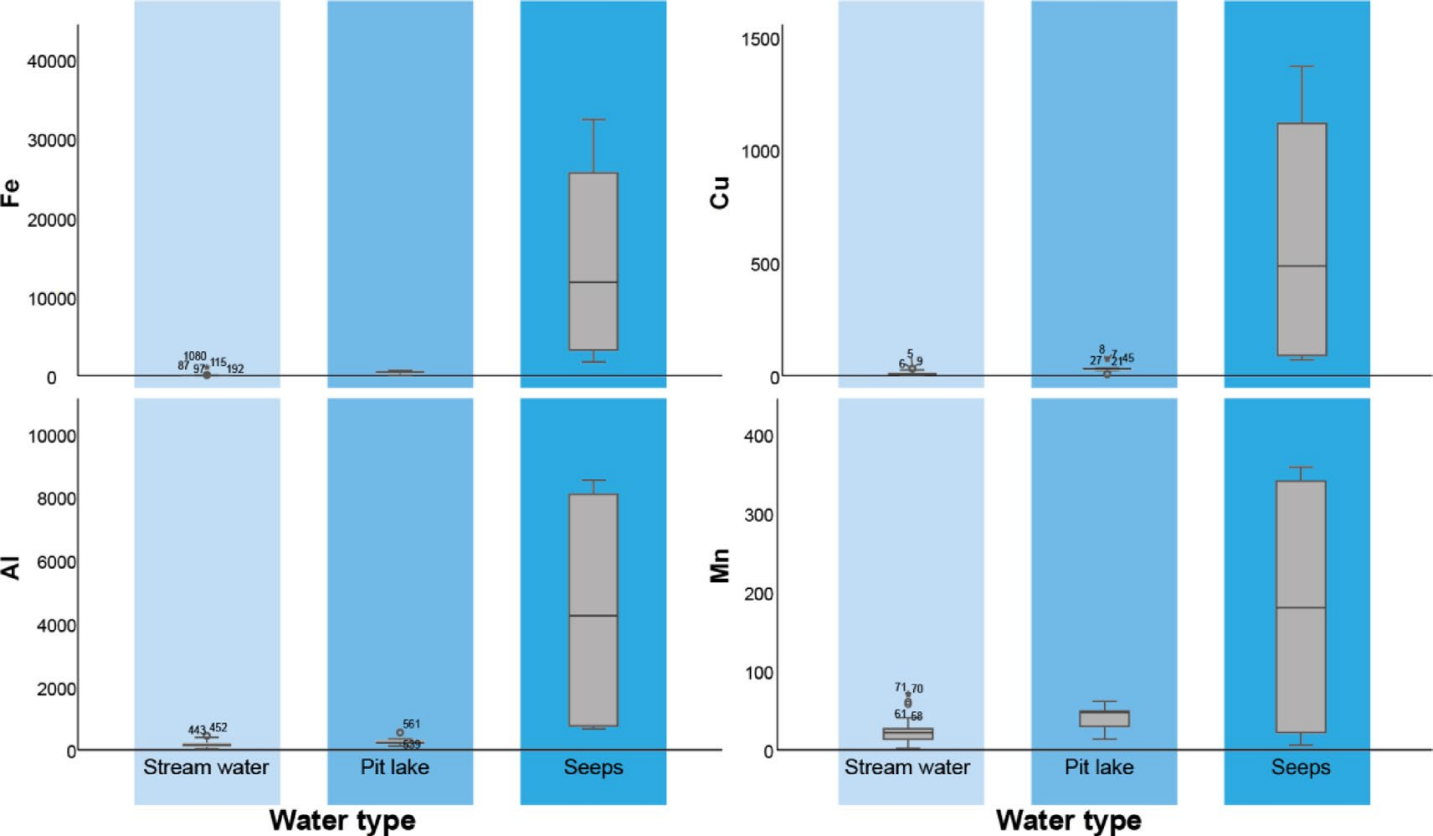
Box plots showing the concentration of Fe, Al, Mn, and Cu in the different types of water (stream water, pit lake water, and seeps).

Pit lakes, due to prolonged residence times and a balance of redox and hydrological inputs, display relatively stable PTE concentrations (Fig. 3). Similar observations were made by Santofimia^51^ and López-Pamo^52^. Consequently, the concentrations of PTE in this system remain within the same range across different sampling campaigns, reflecting the confined nature of the system and its capacity to maintain a steady range of values over time. In contrast, seeps are characterized by high reactivity and geochemical flux. These seeps function as PTE accumulation zones, likely due to the limited water volume in direct contact with reactive materials for short periods, which prevents rapid infiltration, evaporation, or the precipitation of secondary minerals that might contribute to reducing the water contaminant load.

**Fig. 4:**
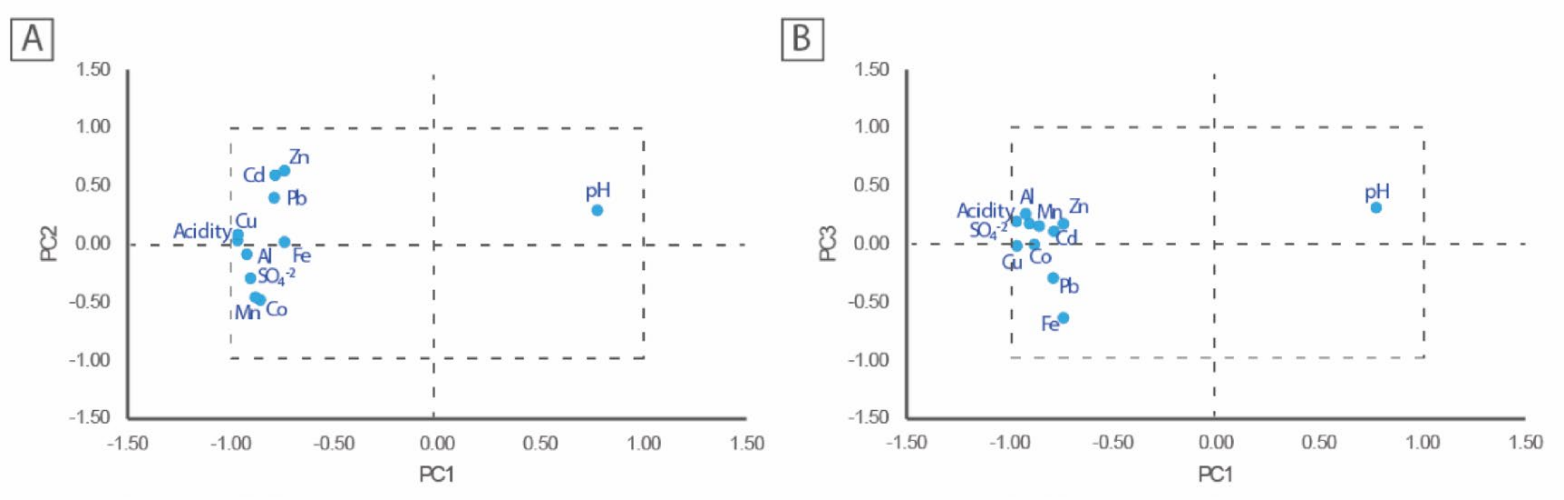
Distribution of the different parameters in the space of the three principal components: (**A**) PC1 and PC2; (**B**) PC1 and PC3.

The correlation matrix (Table S1) revealed strong positive associations between acidity and metals such as Cu, Al, Mn, Zn, Fe, and As, suggesting their involvement in acidity generation. Cu and Al are particularly important in acid-generating hydrolysis reactions. Mn and Zn, while generally less hydrolytic, may contribute under extreme acidic conditions through complexation and redox reactions. The results further indicate that higher acidity, representing the combined presence of free protons and hydrolyzable metal ions, is associated with lower pH values (*r* = − 0.82, Tabel S1) and reduced K concentrations (*r* = − 0.40, Table S1). PCA analysis (Fig. 4) identified three principal components grouping variables influenced by acidity. The first component, comprising acidity, Al, Fe, Cu, and sulfate, reflects the dominant geochemical processes in AMD systems, namely sulfide mineral oxidation and subsequent metal hydrolysis. The second and third components show that Mn-Co and Zn-Cd-Pb are also governed by acid-driven mechanisms, likely through co-precipitation or adsorption onto iron and aluminum hydroxides. The strong negative correlation between pH and these metals further supports the role of acidity in enhancing their mobility and persistence in solution. LDA (Table 2, Figure S1) correctly classified 98% of water samples, demonstrating high discriminatory power using key hydrochemical indicators. The rows represent the actual classification, while the columns indicate the classifications assigned by the LDA. The variables most significant in group discrimination are EC, sulfate, pH, As, Mn, K, Zn, Ca, Pb, and Cu. The leave-one-out classification matrix (Table 2) shows that stream water and water seeps samples were correctly classified, with only one sample from the pit lake group being misclassified. The high level of discrimination is clearly illustrated in Figure S1, where the water seeps are completely separated from the other two water types. Moreover, there is minimal overlap between the samples collected from stream water and pit lakes.

**Table 2:** Sample classification matrix for the Stepwise linear discriminant analysis (LDA).

	Stream water	Pit lake	Seeps	Total
Stream water	31	0	0	31
Pit lake	1	14	0	15
Seeps	0	0	4	4

Due to the limited number of samples obtained from seeps (*n* = 4) and the results of the discriminant analysis, samples were removed from the data set. Within the total dataset, they can be considered outliers due to their significant divergence. Therefore, the model’s effectiveness may be site-specific due to the limited sample size and unique geochemical setting. In particular, the exclusion of seeps from the dataset due to their outlier behavior could reduce model generalizability. Nevertheless, it supports the assumption that acidity estimation represents a significant challenge in these complex environments, requiring a site-specific approach.

PCA was applied to the subset of 46 samples retained after outlier removal (see Sect. "Model development"), with the aim of identifying any associations between acidity and the other parameters. To facilitate the comprehension of the geometric relationships between the variables, Fig. 4 presents the loading plots derived from the PCA. The first two principal components have eigenvalues above one, representing approximately 86% of the total variance. However, the decision was made to retain the first three axes, which together account for 94% of the total variance (PC1, PC2, PC3). Although PC3 has an eigenvalue slightly below 1 (Table S2), it revealed relevant structure in the data, particularly by clarifying associations between acidity (our target variable) and specific groups of variables not fully resolved in the first factorial plane (PC1 vs. PC2).These geometrical relationships can be easily observed from biplots (or factorial planes), which are illustrated in Fig. 4.

The first factorial plane (PC1/PC2), accounting for ~ 86% of the total variance, Table S2) shows three groups of highly correlated variables: acidity, Al, Fe, Cu, and sulfate; Mn and Co; Pb, Cd, and Zn (Fig. 4A). These variables exhibit a strong association, indicating that a shared underlying factor governs their concentrations in the water samples. All these variables show strong positive correlations with PC1. In contrast, pH shows a strong negative correlation with PC1 and is negatively correlated with Al and, to some extent, with acidity, Cu and Fe (Fig. 4A). This inverse relationship between pH and acidity, as well as other PTE concentrations, reflects the well-known phenomenon where lower pH values (higher acidity) correspond to higher solubility and mobility of PTE in water. The second factorial plane (PC1/PC3), explaining approximately 79% of the total variance (Table S2), reveals a negative correlation between Fe and pH. It also shows a distinct grouping of acidity with sulfate, Al, Mn, Zn, Cd, Cu, Co, and, to a lesser extent, Pb, suggesting a common geochemical control over their distribution. (Fig. 4B). Overall, the biplot on the left-hand side associates water acidity with Fe-Al oxyhydroxides containing Cu, while the biplot on the right-hand side indicates that PTE-bearing Al-Mn oxyhydroxides also contributes to the acidity of the water samples. The inverse relationship between pH and PTE concentrations highlights the potential of these parameters to serve as AMD indicators and consequent impacts, with implications for monitoring and remediation strategies.

Given that the stepwise LDA model indicated that acidity was not a significant discriminating factor in the classification of water types, stepwise multiple linear regression (MLR) was employed to identify the hydrochemical variables that most accurately predict acidity concentrations in water samples affected by AMD. The resulting linear models are presented in Table 3. The coefficient of determination (R²) indicates the proportion of variance explained by each regression model, which improves with the addition of each new variable. All five regression models are statistically significant (ρ < 0.005), with a Durbin-Watson statistic of 1.64 (in the range of 1.5 to 2.5), indicating the absence of significant autocorrelation in the residuals, a crucial condition for model validation^53^.

Stepwise MLR modeling identified Cu as the most influential predictor of acidity (R² ~95%), followed by Al, pH, Mn, and Zn. These results underscore the importance of redox-sensitive metals in controlling proton activity in AMD. The final model (M5) achieved an R² of 99%, with a Durbin-Watson statistic of 1.64, indicating no significant autocorrelation.

**Table 3:** Linear regression models used to predict water acidity from hydrochemical parameters in extremely acidic environments.

Model	Equation	*R* ^2^
M1	Acidity = 466.760 + 86.057 x [Cu]	94.5%
M2	Acidity = 33.627 + 57.721 x [Cu] + 4.447 x [Al]	96.4%
M3	Acidity = -2454.827 + 60.425 x [Cu] + 5.268 x [Al] + 848.190 x [pH]	96.8%
M4	Acidity = -3233.291 + 58.342 x [Cu] + 3.820 x [Al] + 1092.488 x [pH] + 15.453 x [Mn]	97.2%
M5	Acidity = -1260.085 + 12.282 x [Cu] -3.652 x [Al] + 496.005 x [pH] + 68.542 x [Mn] + 7.068 x [Zn]	99.2%

**Fig. 5:**
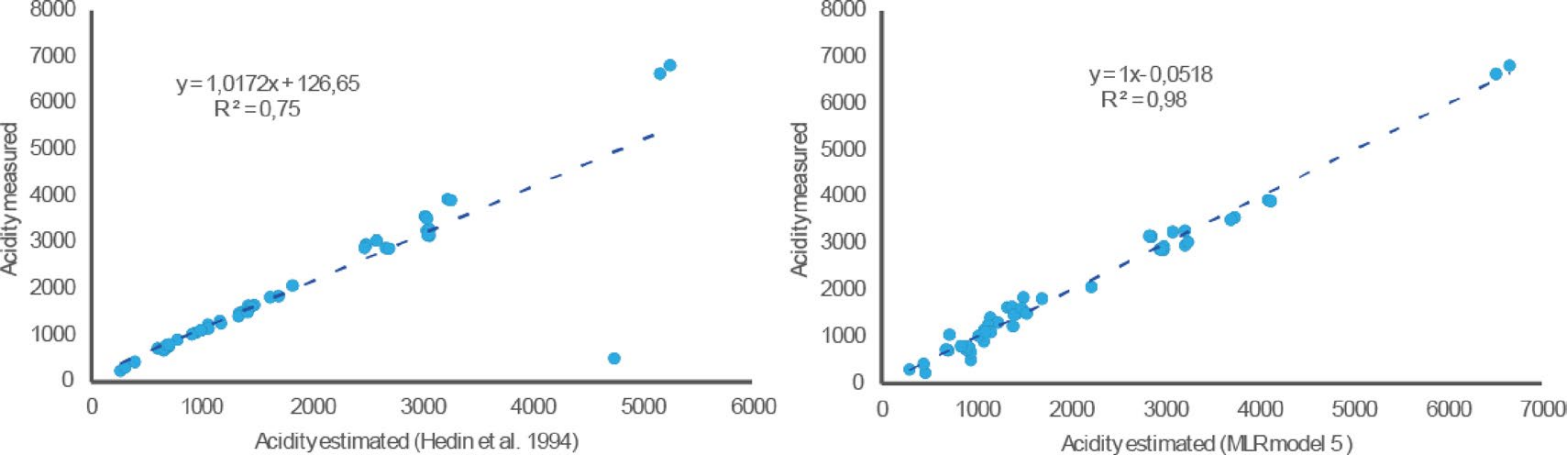
Scatter plots showing the correlation between measured acidity and acidity estimated by the two methods: conventional model (left-hand side) and MLR model (right-hand side).

To validate the obtained MLR model compared to the conventional method described by Hedin^20^, the mean absolute error (MAE) and the mean absolute percentage error (MAPE) were calculated for both approaches. The conventional method, which includes iron speciation along with Al, Mn, and pH, yielded a MAE of 344 and a MAPE of 29%. In contrast, the MLR model produced a significantly lower MAE of 151 and a MAPE of 13%. Comparison with the conventional method^20^ showed that the MLR model provided lower MAE (151 vs. 344) and MAPE (13% vs. 29%), confirming improved accuracy (Fig. 5). However, limitations include potential overfitting due to stepwise selection, the modest sample size (*n* = 46), and site-specific behavior, which may limit extrapolation to other AMD sites. Future studies should validate the model with independent datasets and explore additional parameters such as speciation or mineralogical context. Overall, this study emphasizes the mechanistic linkage between acidity and metal mobilization in AMD systems, particularly the roles of Cu, Al, Mn, and Zn. These findings provide valuable insight for refining predictive models and tailoring monitoring programs in contaminated mining environments.

## Conclusion

The Trimpancho mining complex, representative of legacy mining sites in the IPB, exhibits pronounced features of AMD, including extremely low pH (often < 3), high sulfate concentrations, and elevated concentrations of PTE. Seasonal field campaigns revealed significant spatiotemporal variability in hydrochemical composition, largely driven by precipitation and surface water dynamics. For example, rainfall events were observed to temporarily dilute metal concentrations, emphasizing the strong coupling between climatic variability and AMD chemistry.

The application of linear discriminant analysis successfully differentiated between hydrochemical environments (stream waters, pit lakes, and seeps) each with distinct contamination profiles. Stream waters reflect flow-regulated dispersion patterns, while seeps act as localized PTE accumulation zones, showing transient yet highly concentrated pollutant levels.

Multivariate analysis confirmed that acidity is closely linked to the hydrolysis of redox-sensitive metals. PCA identified Fe, Al, Cu, and sulfate as dominant contributors to acidity generation. These findings validate the use of acidity, pH, and selected PTEs as reliable indicators for AMD impact assessment.

The MLR model developed in this study, particularly the optimized M5 version, demonstrated superior predictive performance (R² = 0.99), significantly outperforming conventional estimation approaches. Cu emerged as the single most influential predictor of acidity, highlighting its central role in AMD geochemistry. These results reinforce the need for site-specific, data-driven models to effectively capture the geochemical complexity of AMD systems.

This research advances our understanding of how AMD evolves under dynamic environmental conditions and provides a framework for predictive monitoring. By integrating seasonal variability, multivariate statistics, and field-based observations, the study offers actionable insights for environmental management. Future work should aim to validate the model across additional mining sites and under varying hydrological regimes to improve its generalizability.

Ultimately, the study highlights the importance of tailoring remediation and monitoring strategies to the specific hydrogeochemical and climatic context of each affected site. Addressing AMD requires not only mechanistic understanding but also interdisciplinary approaches that bridge geochemistry, hydrology, and environmental policy—key tenets of sustainable mine site restoration.

## Supplementary Information

Below is the link to the electronic supplementary material.

Supplementary Material 1

## Data Availability

Data are available upon reasonable request from Ana Barroso (id9873@uminho.pt).
