# Supplementary material for: A data-driven high-accuracy modelling of acidity behavior in heavily contaminated mining environments
Source: Sci Rep. 2025 Sep 30;15:34043. doi: 10.1038/s41598-025-14273-9 (PMC12484933; doi:10.1038/s41598-025-14273-9)
Supplement: Supplementary file 1 — Supplementary Material 1 [file 41598_2025_14273_MOESM1_ESM.docx]

**Article title:**

A Data-driven High-Accuracy Modelling of Acidity Behavior in Heavily Contaminated Mining Environments

**Supplementary material**


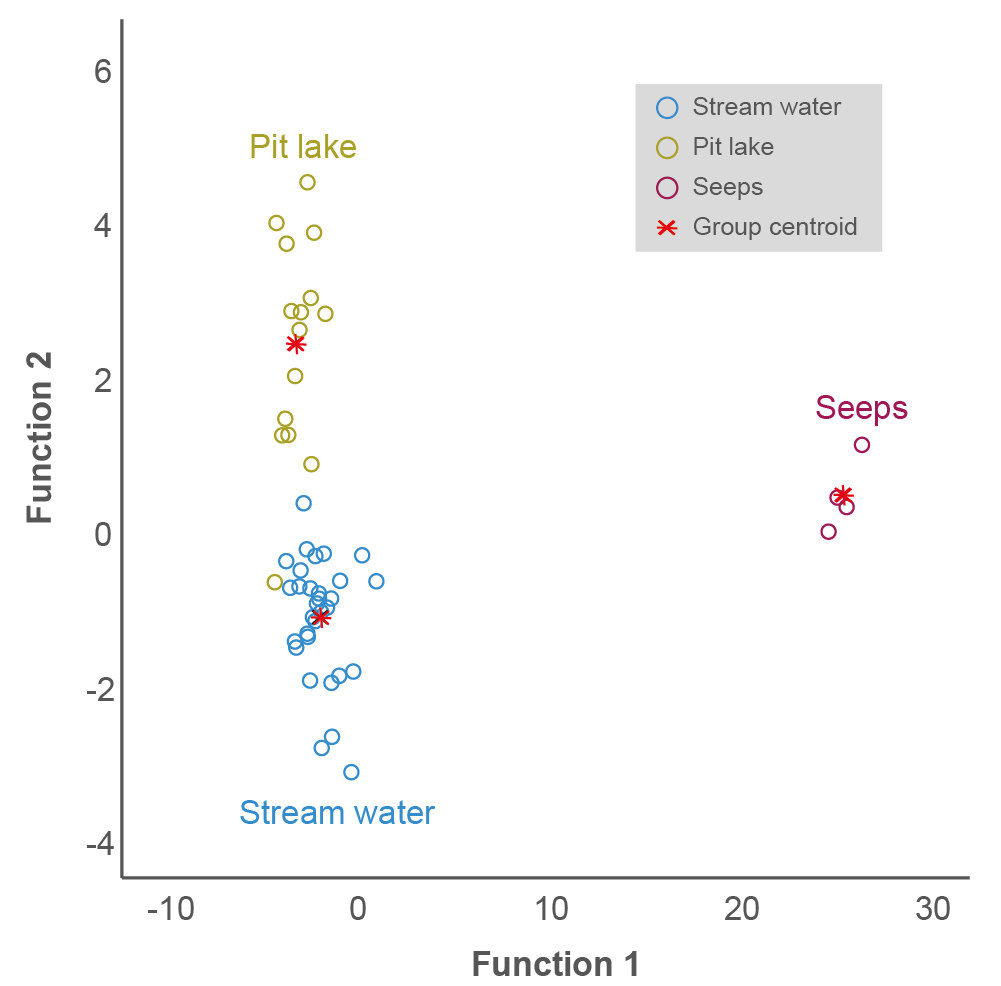


**Figure S1.** Combined-group plot resulting from canonical discriminant functions.

**Table S1.** Spearman correlation matrix for measured parameters.

|  | **Acidity** | **pH** | **EC** | **Sulfate** | **Fe**_(total)_ | **Fe^2+^** | **Fe^3+^** | **Al** | **As** | **Cd** | **Ca** | **Cu** | **K** | **Mg** | **Mn** | **Co** | **Zn** | **Pb** |
| --- | --- | --- | --- | --- | --- | --- | --- | --- | --- | --- | --- | --- | --- | --- | --- | --- | --- | --- |
| **Acidity** | 1 |  |  |  |  |  |  |  |  |  |  |  |  |  |  |  |  |  |
| **pH** | -0,82** | 1 |  |  |  |  |  |  |  |  |  |  |  |  |  |  |  |  |
| **EC** | 0,86** | -0,93** | 1 |  |  |  |  |  |  |  |  |  |  |  |  |  |  |  |
| **Sulfate** | 0,98** | -0,81** | 0,88** | 1 |  |  |  |  |  |  |  |  |  |  |  |  |  |  |
| **Fe**_(total)_ | 0,77** | -0,78** | 0,81** | 0,78** | 1 |  |  |  |  |  |  |  |  |  |  |  |  |  |
| **Fe^2+^** | 0,74** | -0,60** | 0,63** | 0,76** | 0,88** | 1 |  |  |  |  |  |  |  |  |  |  |  |  |
| **Fe^3+^** | 0,77** | -0,79** | 0,81** | 0,78** | 1** | 0,88** | 1 |  |  |  |  |  |  |  |  |  |  |  |
| **Al** | 0,92** | -0,85** | 0,91** | 0,89** | 0,76** | 0,62** | 0,76** | 1 |  |  |  |  |  |  |  |  |  |  |
| **As** | 0,67** | -0,68** | 0,65** | 0,64** | 0,82** | 0,74** | 0,82** | 0,65** | 1 |  |  |  |  |  |  |  |  |  |
| **Cd** | 0,83** | -0,75** | 0,80** | 0,80** | 0,70** | 0,57** | 0,70** | 0,85** | 0,81** | 1 |  |  |  |  |  |  |  |  |
| **Ca** | 0,78** | -0,72** | 0,81** | 0,83** | 0,56** | 0,49** | 0,57** | 0,72** | 0,37** | 0,61** | 1 |  |  |  |  |  |  |  |
| **Cu** | 0,88** | -0,82** | 0,87** | 0,88** | 0,82** | 0,76** | 0,82** | 0,86** | 0,77** | 0,84** | 0,67** | 1 |  |  |  |  |  |  |
| **K** | -0,40** | 0,33* | -0,30* | -0,38** | -0,17 | -0,18 | -0,17 | -0,32* | -0,08 | -0,21 | -0,34* | -0,27 | 1 |  |  |  |  |  |
| **Mg** | 0,79** | -0,73** | 0,83** | 0,83** | 0,60** | 0,53** | 0,60** | 0,73** | 0,33* | 0,58** | 0,89** | 0,66** | -0,38** | 1 |  |  |  |  |
| **Mn** | 0,84** | -0,76** | 0,87** | 0,87** | 0,66** | 0,57** | 0,66** | 0,82** | 0,48** | 0,72** | 0,90** | 0,77** | -0,44** | 0,94** | 1 |  |  |  |
| **Co** | 0,90** | -0,87** | 0,96** | 0,92** | 0,79** | 0,67** | 0,79** | 0,91** | 0,62** | 0,80** | 0,85** | 0,87** | -0,36* | 0,90** | 0,95** | 1 |  |  |
| **Zn** | 0,52** | -0,32* | 0,35* | 0,43** | 0,10 | 0,06 | 0,10 | 0,57** | 0,28 | 0,66** | 0,37** | 0,36* | -0,27 | 0,34* | 0,50** | 0,42** | 1 |  |
| **Pb** | 0,76** | -0,74** | 0,74** | 0,74** | 0,77** | 0,67** | 0,77** | 0,77** | 0,83** | 0,88** | 0,56** | 0,75** | -0,23 | 0,52** | 0,63** | 0,73** | 0,51** | 1 |
| **Note:** Significant level ⍴ = 0.01** and ⍴ = 0.05* | | | | | | | | | | | | | | | | | | |

**Table S2.** Loadings of variables and explained variance (individual and cumulative) of principal components for the dataset.

|  | PC1 | PC2 | PC3 |
| --- | --- | --- | --- |
| Eigenvalue | 7.99 | 1.54 | 0.79 |
| Exp variance (%) | 72.63 | 14.01 | 7.22 |
| Cumulative variance (%) | 72.63 | 86.64 | 93.86 |
| pH | 0.78 | 0.29 | 0.32 |
| Acidity | -0.97 | 0.03 | 0.20 |
| SO4-2 | -0.90 | -0.29 | 0.18 |
| Fe_(total)_ | -0.74 | 0.02 | -0.63 |
| Al | -0.92 | -0.09 | 0.26 |
| Cd | -0.79 | 0.59 | 0.11 |
| Cu | -0.97 | 0.09 | -0.01 |
| Mn | -0.86 | -0.48 | 0.16 |
| Co | -0.88 | -0.45 | 0.00 |
| Zn | -0.74 | 0.63 | 0.18 |
| Pb | -0.79 | 0.40 | -0.29 |
